# Indicators of implicit and explicit social anxiety influence threat-related interpretive bias as a function of working memory capacity

**DOI:** 10.3389/fnhum.2013.00220

**Published:** 2013-05-23

**Authors:** Elske Salemink, Malte Friese, Emily Drake, Bundy Mackintosh, Laura Hoppitt

**Affiliations:** ^1^Department of Developmental Psychology, Cognitive Science Centre Amsterdam, University of AmsterdamAmsterdam, Netherlands; ^2^Department of Psychology, Saarland UniversitySaarbruecken, Germany; ^3^School of Medicine, Health Policy and Practice, University of East AngliaNorwich, UK; ^4^School of Social Work and Psychology, University of East AngliaNorwich, UK

**Keywords:** threat-related interpretive bias, dual process model, working memory capacity, anxiety

## Abstract

Interpretive biases play a crucial role in anxiety disorders. The aim of the current study was to examine factors that determine the relative strength of threat-related interpretive biases that are characteristic of individuals high in social anxiety. Different (dual process) models argue that both implicit and explicit processes determine information processing biases and behavior, and that their impact is moderated by the availability of executive resources such as working memory capacity (WMC). Based on these models, we expected indicators of implicit social anxiety to predict threat-related interpretive bias in individuals low, but not high in WMC. Indicators of explicit social anxiety should predict threat-related interpretive bias in individuals high, but not low in WMC. As expected, WMC moderated the impact of implicit social anxiety on threat-related interpretive bias, although the simple slope for individuals low in WMC was not statistically significant. The hypotheses regarding explicit social anxiety (with fear of negative evaluation used as an indicator) were fully supported. The clinical implications of these findings are discussed.

## Introduction

Sarah is talking to someone and that person suddenly starts to yawn. She immediately thinks that she is telling a boring story and that she is a dead loss as a storyteller. Negatively biased interpretations of ambiguous social events such as this one by Sarah are known to be characteristic of individuals high in social anxiety (Mathews and Mackintosh, 1998). It has been argued that both for theoretical and clinical reasons, it is important to understand the mechanisms underlying this threat-related interpretive bias (Blanchette and Richards, 2010). Therefore, the aim of the current study was to examine the role of both explicit and implicit anxiety, and regulatory control processes in relation to this threat-related interpretive bias.

Dual process models propose that behavioral responses are the consequence of two different types of processes; implicit and explicit processes (e.g., Strack and Deutsch, 2004). These models have recently been applied to psychopathology (anxiety: Ouimet et al., 2009; depression: Beevers, 2005). While specific descriptions vary, it has been argued that implicit processes are based on automatic associations of concepts in memory and more explicit processes are characterized by more propositional knowledge. Importantly, it is assumed that the relative impact of these processes depends on the availability of control resources, for example dispositional factors such as working memory capacity (WMC, Hofmann et al., 2008). The behavior of individuals high in WMC is expected to be more strongly influenced by explicit processes, while behavior of individuals low in WMC is expected to be more strongly influenced by implicit processes. These assumptions have been supported for self-regulatory behaviors in domains such as aggression, food consumption, and sexual interest behavior (Hofmann et al., 2008). For example, automatic attitudes on eating predicted actual candy eating in participants with low WMC, but not in participants with high WMC. The opposite pattern was observed for more explicit attitudes on eating; this predicted the amount of candy consumed only in individuals with high WMC (Hofmann et al., 2008).

Specific models in the field of anxiety argue that processing biases can also be conceptualized as the joint outcome of an interaction between automatic tendencies and control over these tendencies. Mathews and Mackintosh (1998), for example, proposed a model in which threat-related biases in information processing depend on activation of a more automatic threat-detection system and a top-down regulatory control system. Biases in information processing are predicted to be present when the activation of the affective system exceeds the capacity for control over (mental) contents (see also Mathews and MacLeod, 2005). Neurobiological data suggest that threat-related information processing might be related to increased amygdala activity coupled with a decrease in the recruitment of prefrontal control mechanisms (Blanchette and Richards, 2010). Derryberry and Reed (2002) provided empirical support for such claims regarding threat-related attentional bias; anxious individuals with low levels of regulatory control had stronger threat-related attentional biases than anxious individuals with high levels of regulatory control (comparable findings have been observed for alcohol-related attentional bias; Friese et al., 2010). Less is known regarding such an interaction in threat-related interpretive bias.

The aim of the current study is to examine whether the expression of threat-related interpretive bias would arise from a similar interaction between anxiety and regulatory control. While threat-related interpretive biases have been studied for decades, little research has investigated the psychological processes that determine the strength of such biases. We plan to fill this gap by building on the outlined dual-process frameworks. We made a distinction between explicit and implicit indices of social anxiety as research has shown that these indices explain additional variance in anxiety and are differentially related to aspects of anxiety-related (psychopathological) behavior (Egloff and Schmukle, 2002; Glashouwer and De Jong, 2010). In a series of studies, Egloff and Schmukle showed that implicit indicators of anxiety (automatic associations of the self with anxiety) and explicit indicators of anxiety (deliberate judgments of the self as anxious) functioned in a complementary manner. For example implicit indicators predicted change in performance after stress that explicit indicators were unable to predict. In the current study, fear of negative evaluation was used as an indicator of explicit social anxiety as it is considered a core feature of social anxiety (Rapee and Heimberg, 1997) and often used in research [for a meta-analysis see Acarturk et al. (2009)]. Dual-process theories propose that implicit processes impact stronger on indices of outcome behavior in individuals with low regulatory control but not high in regulatory control as individuals with high regulatory control are expected to have enough capacity to override the influence of the automatic system. In the current context of anxiety, it has been suggested that “… anxious individuals find attending to threatening stimuli distressing and consequently try to avoid them … ” (Mathews and Mackintosh, 1998, p. 546) and individuals with high regulatory control might be better in achieving that. Conversely, it is proposed that explicit processes impact stronger on behavior in individuals with high levels of control. We expected based on dual process models and earlier findings regarding attentional bias, that indicators of implicit social anxiety (Egloff and Schmukle, 2002; Westberg et al., 2007) predict threat-related interpretive bias for individuals low, but not high in WMC. Conversely, indicators of explicit social anxiety predict interpretive bias for individuals high, but not low in WMC. These hypotheses postulate a dynamic interplay of different psychological processes interacting to determine the strength of threat-related interpretive biases. They thereby go beyond the assumption of main effects (i.e., stronger social anxiety leads to a stronger interpretive bias) by distinguishing between the differential influences of implicit and explicit indicators of social anxiety and identifying the boundary conditions when they will be more or less influential in impacting upon interpretive biases. Support for these assumptions would provide novel and unique evidence for the psychological processes underlying the expression of threat-related interpretive biases and how they interact in determining the magnitude of these biases.

## Methods^1^

### Participants

A total of 79 participants aged between 18 and 35 years were recruited from the University of East Anglia via posters and online advertisements regarding the effects of emotion on comprehension of information. One participant inadvertently completed the IAT twice and because the first assessment data were overwritten by the second, the data were excluded. Two further participants were excluded due to high error rates on the operation span task. Finally, preparatory regression analyses revealed four multivariate outliers (based on studentized deleted residuals and mahalanobis distance) who were excluded from the analyses. The final sample consisted of 72 participants and the mean age was 23.64 years (*SD* = 4.16, 49 females). Participants were either entered into a prize draw or received £8 to compensate for their time.

### Materials

#### Implicit association test (IAT)

An IAT containing self and social anxiety related words was used as an indicator of implicit social anxiety (Egloff and Schmukle, 2002; Westberg et al., 2007). Participants had to classify stimuli from four categories using two response keys; one categorization concerned *self* vs. *others* and the second concerned *social anxiety* vs. *relaxed*. The IAT consisted of seven blocks. During the first block, participants practiced categorizing stimuli into the *self* or *others* categories (20 trials) and in the second block into the *social anxiety* or *relaxed* categories (20 trials). In the third and fourth block (combination blocks), participants classified stimuli into all categories simultaneously (20 trials and 60 trials, respectively). Participants pressed one key when stimuli referred to either *self* or *social anxiety* and another key when they referred to *others* or *relaxed*. In the fifth block (40 trials), the categories *social anxiety* and *relaxed* changed sides resulting in opposite response assignments. In the sixth and seventh block (reversed combination blocks), participants again categorized all categories simultaneously (20 trials and 60 trials respectively). An IAT-index was calculated using the D600 improved scoring algorithm (Greenwald et al., 2003). Following the formula presented by Greenwald et al., all combination blocks were included (blocks 3, 4, 6, and 7), error penalties (600 ms) were given, and results were standardized at the level of the participant. The D600 measure was calculated so that higher scores reflect stronger associations between “self” and “social anxiety” as compared to “self” and “relaxed.” It thus provides a relative measure of the implicit association between self and social anxiety. Previous research has demonstrated that the anxiety IAT exhibits good internal consistency (Cronbach's alphas in the range of 0.80) and predicts behavioral indicators of anxiety (Egloff and Schmukle, 2002).

#### Fear of negative evaluation (FNE)

The FNE scale measures fear of being evaluated negatively by others and was used as an indicator of explicit social anxiety (Watson and Friend, 1969). It comprises 30 statements (e.g., I rarely worry about seeming foolish to others), asking participants to rate each item as either true or false. The FNE has alpha coefficients of 0.94 (student population, Watson and Friend, 1969; clinical population, Oei et al., 1991), indicative of high internal consistency.

#### Complex operation span task (OSPAN)

The OSPAN is a widely used complex operation span task, providing a measure of individual differences in WMC (Unsworth et al., 2005). Participants were presented with a set of equations on the screen consisting of one addition or subtraction and a multiplication [e.g., (2 × 4) − 3 = 5]. They were asked to indicate whether the presented result was true or false. Then a letter was presented and participants remembered the letters in the order in which they appeared. Feedback was provided regarding the number of correctly solved equations and letters recalled. The program started with a practice phase consisting of practicing letter recall, math portions, and their combination respectively. In the assessment phase, participants received three trials of each set size, with set sizes ranging from three to seven. Order of set sizes was random for each participant. An 85% accuracy criterion on the math operations was required for all the participants to ensure that they were not trading off between solving the operations and remembering the words (Unsworth et al., 2005). A WMC index was computed by summing up the number of correctly recalled sets. This index has both good internal consistency (alpha = 0.78) and test-retest reliability (0.83), and was correlated with other WM span measures and with a factor composed of fluid abilities measures (Unsworth et al., 2005).

#### Word sentence association paradigm (WSAP)

The WSAP provides an assessment of threat-related interpretive bias (Beard and Amir, 2008, 2009). On each trial, a word was presented for 500 ms, followed by a sentence. For half of the trials, the word and sentence facilitated a threat-related interpretation (e.g., embarrassing—People laugh after something you said), and on the other half a non-threat-related interpretation (e.g., funny—People laugh after something you said). Participants indicated whether the word and sentence were related by pressing a “Yes” or “No” key. Seventy-six sentences describing social situations were selected from those used by Beard and Amir (2008, 2009). Each sentence was once paired with a threat-related and once with a non-threat-related word. These 152 word-sentence pairs were divided into two sets and participants were randomly assigned to a set. An interpretive bias index was calculated by subtracting the percentage of non-threat-related endorsements from the percentage of threat-related endorsements and higher scores represent a stronger threat-related interpretive bias. Previous research has revealed that both threat endorsements and non-threat endorsements were significantly correlated with level of social anxiety (Beard and Amir, 2008, 2009).

### Procedure

Participants received an information sheet and provided informed consent. Next, participants completed the IAT, the OSPAN, and the WSAP on the computer before completing the FNE scale using paper and pencil. Finally, participants were debriefed and given the opportunity to ask questions. The testing session lasted approximately 60 min.

## Results

To examine the relationship between indicators of implicit and explicit social anxiety, WMC, and threat-related interpretive bias, zero-order correlations were computed between these variables (see Table 1 for means, standard deviations, and correlations). Explicit social anxiety, as indicated by the FNE scale, was positively associated with threat-related interpretive bias and implicit social anxiety, as indicated by the IAT, correlated positively with WMC. These zero-order correlations should, however, be interpreted in the context of the multiple regression analyses reported next.

**Table 1:** **Correlations between indicators of implicit and explicit social anxiety, WMC, and threat-related interpretive bias, (*n* = 72)**.

	**1.**	**2.**	**3.**	**4.**
1. Implicit social anxiety (indicated by the IAT)	−			
2. Explicit social anxiety (indicated by the FNE)	−0.05	−		
3. Working memory capacity (indicated by the OSPAN)	0.44^**^	−0.06	−	
4. Threat-related interpretive bias (indicated by the WSAP)	0.04	0.24^*^	0.10	−
*M*	−0.15	16.8	40.7	−20.9
*SD*	0.31	6.9	14.6	15.4

*Note*:

* p < 0.05,

** p < 0.01. IAT, implicit association test; FNE, fear of negative evaluation scale; OSPAN, complex operation span task; WSAP, word sentence association paradigm.

**Figure 1:**
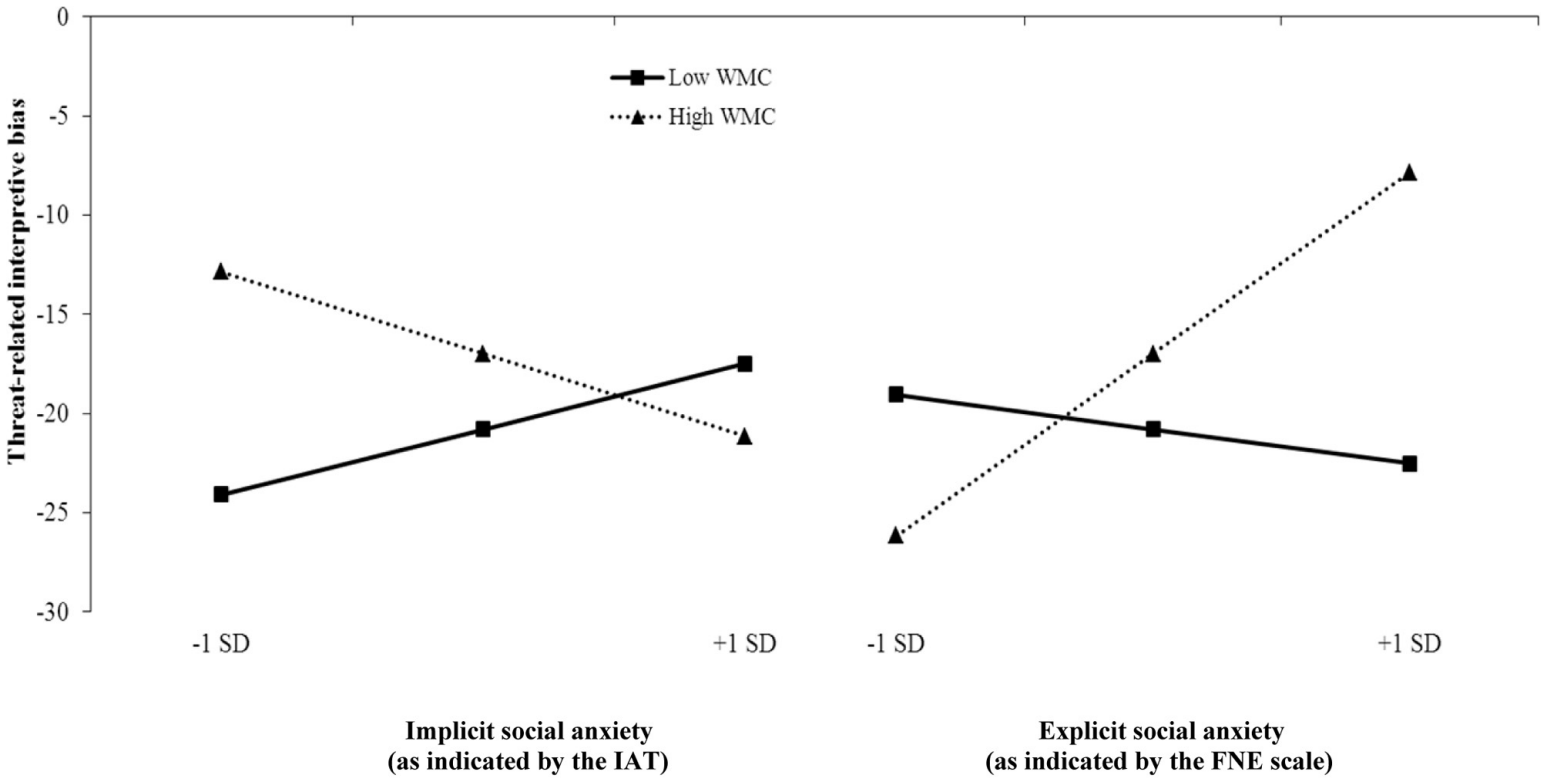
**Influence of indicators of implicit and explicit social anxiety on threat-related interpretive bias as a function of working memory capacity (WMC).** The graph shows predicted interpretive bias scores (not standardized for illustrative purposes) for individuals with low (−1 SD) and high (+1 SD) implicit social anxiety, as indicated by the IAT, (left panel) or individuals with low (−1 SD) and high (+1 SD) fear of negative evaluation as an indicator of explicit social anxiety (right panel) depending on low (−1 SD) and high (+1 SD) working memory capacity.

In order to investigate whether WMC moderates the impact of implicit and explicit indicators of social anxiety on threat-related interpretive bias, we performed a moderated regression analysis on interpretive bias as the dependent variable. To reduce multicollinearity and to arrive at the correct beta weights, all variables were first z-standardized (Aiken and West, 1991). As predictors, we entered implicit social anxiety (indicated by the IAT), explicit social anxiety (indicated by the FNE scale), WMC, and the interactions between implicit social anxiety and WMC, and explicit social anxiety and WMC^2^. The regression analysis [*R*^2^ = 0.22, *F*_(5, 71)_ = 3.76, *p* = 0.005] yielded three significant predictors; explicit social anxiety, β = 0.24, *p* = 0.029, and the predicted interaction effects of implicit social anxiety × WMC, β = −0.24, *p* = 0.048, and explicit social anxiety × WMC, β = 0.35, *p* = 0.007. Consistent with previous studies, high scores on fear of negative evaluation (as an indicator of explicit social anxiety) were associated with a stronger threat-related interpretive bias. The interaction effects are depicted in Figure 1 (left: implicit social anxiety, as indicated by the IAT; right: explicit social anxiety, as indicated by the FNE scale). As expected, the significant interaction between implicit social anxiety and WMC appears to indicate that IAT scores, as an indicator of implicit social anxiety, were positively associated with threat-related interpretive bias for individuals low in WMC, with an opposite pattern of effects for participants high in WMC. However, although the interaction revealed the expected moderating effect of WMC, simple slope analyses were not significant for either those low, β = 0.21, *p* = 0.195, or high in WMC β = −0.27, *p* = 0.135. Regarding the explicit social anxiety (as indicated by the FNE scale) × WMC interaction, simple slope tests confirmed the hypothesis that explicit social anxiety as indicated by the FNE scale predicted interpretive bias for individuals high (β = 0.60, *p* < 0.001), but not low in WMC (β = −0.11, *p* = 0.522).

## Discussion

The present study drew on contemporary dual-process models (Mathews and Mackintosh, 1998; Strack and Deutsch, 2004; Ouimet et al., 2009) to investigate the assumption that the magnitude of threat-related interpretive bias depends on indicators of both implicit and explicit social anxiety and that their relative influences crucially hinge on the availability of control resources such as WMC. As predicted, WMC moderated the impact of the implicit indicator of social anxiety on interpretive bias, with the results suggesting a positive relationship between implicit social anxiety, as indicated by the IAT, and interpretive bias for individuals with low, but not high WMC (though the slope failed to reach significance). The predicted opposite pattern was observed for the indicator of explicit social anxiety; fear of negative evaluation was only associated with threat-related interpretive bias in individuals with high, but not low levels of WMC.

While it has been theoretically argued that threat-related interpretive biases are the joint outcome of two tendencies (Mathews and Mackintosh, 1998), empirical data supporting this claim was lacking. To the best of our knowledge, the present study is the first to provide empirical evidence that threat-related interpretive biases can be conceptualized as the result of an interplay between indicators of implicit and explicit social anxiety on the one hand and WMC on the other hand. More generally, the current findings are consistent with studies in the field of health psychology that revealed that control processes can moderate the impact of implicit and explicit processes on self-regulatory behavior (Hofmann et al., 2008; Friese and Hofmann, 2012) and on biases in information processing (Friese et al., 2010).

The finding regarding the role of implicit social anxiety requires future research as the hypothesized slope in individuals with low WMC was not significant and an unexpected positive correlation between implicit social anxiety and WMC was observed. This might be related to the type of IAT used in the current study. That is, implicit social anxiety was indicated by an social anxiety IAT, which assessed the relative strength of associations between the self and social anxiety. While this measure has been used in other studies examining social anxiety (Egloff and Schmukle, 2002; Westberg et al., 2007), in retrospect, it might have been conceptually different from the processes in social anxiety that we focused on. That is, there seems to be a match in content between the explicit indicator of social anxiety (fear of negative evaluations) and the outcome variable (negative interpretive bias in social situations), while the anxiety IAT seems conceptually different. A social evaluative IAT (see for example Clerkin and Teachman, 2010) might potentially better capture the relevant processes and have a different and potentially stronger impact on threat-related interpretive bias. Additionally, the IAT provides a measure of relative strength of associations and is not an absolute measure. Despite these shortcomings, the IAT revealed the hypothesized interaction with WMC in the prediction of threat-related interpretive bias.

Some other study limitations should be acknowledged. In line with previous research (Acarturk et al., 2009), we used fear of negative evaluation as an indicator of explicit social anxiety. It is important to acknowledge that while fear of negative evaluation is considered a hallmark aspect of social anxiety (Rapee and Heimberg, 1997), both constructs are highly related, but not identical (Weeks et al., 2005). Future research should investigate the generalizability of the present findings by replicating this study using other indicators of explicit social anxiety, for example, the Social Phobia Scale and Social Interaction Anxiety Scale (Mattick and Clarke, 1998) or the Liebowitz Social Anxiety Scale (Liebowitz, 1987). In addition, given the comorbidity between anxiety and depression, it would be important to control for depression in future studies. A more methodological limitation is the task order. All participants completed the tasks in the same order (IAT, OSPAN, WSAP, FNE). While this is consistent with other studies examining moderated predictive validity of implicit measures (Hofmann et al., 2008; Friese et al., 2010; Friese and Hofmann, 2012), we cannot rule out that this order might have influenced the results. For example, the OSPAN could have been perceived as stressful, and potentially especially for anxious individuals, and this might have (differentially) affected subsequent assessments. Also, FNE scores may have been inflated for individuals with higher levels of social anxiety due to priming effects by the IAT and WSAP. Importantly, if existent, such a bias would have had negative effects on the overall validity of the scale and should have made it more unlikely (not more likely) to detect the predicted moderation effect. Finally, we tested the theoretical model in unselected individuals. To investigate the clinical implications of our findings, future studies should test the model in highly-anxious (sub)clinical populations as such individuals are specifically characterized by threat-related biases. In addition, directly comparing clinically and non-clinically anxious individuals would be interesting as it has been suggested that those groups differ in the ability to regulate their information processing biases (Macleod and Rutherford, 1992).

The current findings shed light on the underpinnings of threat-related interpretive bias. They have a range of potentially clinically relevant implications. In addition to recent developments regarding interventions that are designed to directly modify information processing biases (CBM training, Macleod and Mathews, 2012), the current findings reveal potential determinants of threat-related interpretive bias. Changing these determinants might affect information processing, however, as the current study has a correlational design, more research is necessary to examine whether those processes are causal agents. There is promising evidence for each process (implicit processes, explicit processes, and WMC) that changing them might be beneficial. First, it has been shown that implicit associations can be modified using Cognitive Behavior Therapy (CBT, Teachman et al., 2008), but also by performing repeated avoidance responses (Wiers et al., 2011). Second, CBT can also change explicit processes such as self-reported socially anxious feelings (Hofmann et al., 2012). Third, increasing control resources might be beneficial as it would allow an individual to counteract the impact of their implicit processes. Indeed, there are exciting possibilities to directly enhance WMC; either using computerized WM training (Klingberg et al., 2005; but see Owen et al., 2010) or transcranial Direct Current Stimulation (Boggio et al., 2007). Thus, the current study identified three types of processes that were related to interpretive bias and recent findings suggest that each of these processes can be modified and, more importantly, affect symptoms of psychopathology.

In conclusion, individual differences in WMC moderated the association between indicators of both implicit and explicit social anxiety on the one hand, and threat-related interpretive biases on the other hand. These findings have significant theoretical and clinical implications.

### Conflict of interest statement

The authors declare that the research was conducted in the absence of any commercial or financial relationships that could be construed as a potential conflict of interest.

## Footnotes

^1^Only those measures relevant for the current hypotheses are listed here. Participants also completed questionnaires regarding general state and trait anxiety, the IAT stimuli, a lexical decision task and an unvalidated ad-hoc created questionnaire regarding personal goal orientation concerning the handling of social situations in everyday life.

^2^To examine the influence of age on the results, we repeated the regression analysis with age added as an additional predictor. Age was not a significant predictor and the previously significant predictors remained significant.

## References

[B1] Acarturk C. Cuijpers P. Van Straten A. De Graaf R. (2009). Psychological treatment of social anxiety disorder: a meta-analysis. Psychol. Med. 39, 241–254 10.1017/S0033291708003590 18507874

[B2] Aiken L. S. West S. G. (1991). Multiple Regression: Testing and Interpreting Interactions. Thousand Oaks, CA: Sage

[B3] Beard C. Amir N. (2008). A multi-session interpretation modification program: changes in interpretation and social anxiety symptoms. Behav. Res. Ther. 46, 1135–1141 10.1016/j.brat.2008.05.012 18675400 PMC3569034

[B4] Beard C. Amir N. (2009). Interpretation in social anxiety: when meaning precedes ambiguity. Cogn. Ther. Res. 33, 406–415 10.1007/s10608-009-9235-0 20046862 PMC2792932

[B5] Beevers C. G. (2005). Cognitive vulnerability to depression: a dual process model. Clin. Psychol. Rev. 25, 975–1002 10.1016/j.cpr.2005.03.003 15905008

[B6] Blanchette I. Richards A. (2010). The influence of affect on higher level cognition: a review of research on interpretation, judgement, decision making and reasoning. Cogn. Emot. 24, 561–595

[B7] Boggio P. S. Bermpohl F. Vergara A. O. Muniz A. L. Nahas F. H. Leme P. B. (2007). Go-no-go task performance improvement after anodal transcranial DC stimulation of the left dorsolateral prefrontal cortex in major depression. J. Affect. Disord. 101, 91–98 10.1016/j.jad.2006.10.026 17166593

[B8] Clerkin E. M. Teachman B. A. (2010). Training implicit social anxiety associations: an experimental intervention. J. Anxiety Disord. 24, 300–308 10.1016/j.janxdis.2010.01.001 20102788 PMC2838945

[B9] Derryberry D. Reed M. A. (2002). Anxiety-related attentional biases and their regulation by attentional control. J. Abnorm. Psychol. 111, 225–236 10.1037/0021-843X.111.2.225 12003445

[B10] Egloff B. Schmukle S. C. (2002). Predictive validity of an implicit association test for assessing anxiety. J. Pers. Soc. Psychol. 83, 1441–1455 10.1037/0022-3514.83.6.1441 12500823

[B11] Friese M. Bargas-Avila J. Hofmann W. Wiers R. W. (2010). Here's looking at you, bud: alcohol-related memory structures predict eye movements for social drinkers with low executive control. Soc. Psychol. Pers. Sci. 1, 143–151

[B12] Friese M. Hofmann W. (2012). Just a little bit longer: viewing time of erotic material from a self-control perspective. Appl. Cogn. Psychol. 26, 489–496

[B13] Glashouwer K. De Jong P. (2010). Disorder-specific automatic self-associations in depression and anxiety: results of the Netherlands study of depression and anxiety. Psychol. Med. 40, 1101–1111 10.1017/S0033291709991371 19811700

[B14] Greenwald A. G. Nosek B. A. Banaji M. R. (2003). Understanding and using the Implicit Association Test: I. An improved scoring algorithm. J. Pers. Soc. Psychol. 85, 197–216 10.1037/0022-3514.85.2.197 12916565

[B15] Hofmann S. G. Asnaani A. Vonk I. J. J. Sawyer A. T. Fang A. (2012). The efficacy of Cognitive Behavioral Therapy: a review of meta-analyses. Cogn. Ther. Res. 36, 427–440 10.1007/s10608-012-9476-1 23459093 PMC3584580

[B16] Hofmann W. Gschwendner T. Friese M. Wiers R. W. Schmitt M. (2008). Working memory capacity and self-regulatory behavior: toward individual differences perspective on behavior determination by automatic versus controlled processes. J. Pers. Soc. Psychol. 95, 962–977 10.1037/a0012705 18808271

[B17] Klingberg T. Fernell E. Olesen P. J. Johnson M. Gustafsson P. Dahlström K. (2005). Computerized training of working memory in children with ADHD: a randomized, controlled trial. J. Am. Acad. Child Adolesc. Psychiatry 44, 177–186 10.1097/00004583-200502000-00010 15689731

[B18] Liebowitz M. R. (1987). Social phobia. Mod. Probl. Pharmacopsychiatry 22, 141–173 2885745 10.1159/000414022

[B19] Macleod C. Mathews A. (2012). Cognitive Bias Modification approaches to anxiety. Ann. Rev. Clin. Psychol. 8, 189–217 10.1146/annurev-clinpsy-032511-143052 22035241

[B20] Macleod C. Rutherford E. M. (1992). Anxiety and the selective processing of emotional information: mediating roles of awareness, trait and state variables, and personal relevance of the stimulus materials. Behav. Res. Ther. 30, 479–491 1520234 10.1016/0005-7967(92)90032-c

[B21] Mathews A. Mackintosh B. (1998). A cognitive model of selective processing in anxiety. Cogn. Ther. Res. 22, 539–560

[B21a] Mathews A. MacLeod C. (2005). Cognitive vulnerability to emotional disorders. Annu. Rev. Clin. Psychol. 1, 167–195 10.1146/annurev.clinpsy.1.102803.143916 17716086

[B22] Mattick R. P. Clarke J. C. (1998). Development and validation of measures of social phobia scrutiny fear and social interaction anxiety. Behav. Res. Ther. 36, 455–470 10.1016/S0005-7967(97)10031-6 9670605

[B23] Oei T. P. S. Kenna D. Evans L. (1991). The reliability, validity and utility of the SAD and FNE scales for anxiety disorder patients. Pers. Individ. Dif. 12, 111–116

[B24] Ouimet A. J. Gawronski B. Dozois D. J. A. (2009). Cognitive vulnerability to anxiety: a review and an integrative model. Clin. Psychol. Rev. 29, 459–470 10.1016/j.cpr.2009.05.004 19552990

[B25] Owen A. M. Hampshire A. Grahn J. A. Stenton R. Dajani S. Burns A. S. (2010). Putting brain training to the test. Nature 465, 775–778 10.1038/nature09042 20407435 PMC2884087

[B26] Rapee R. M. Heimberg R. G. (1997). A cognitive-behavioral model of anxiety in social phobia. Behav. Res. Ther. 35, 741–756 10.1016/S0005-7967(97)00022-3 9256517

[B27] Strack F. Deutsch R. (2004). Reflective and impulsive determinants of social behaviour. Pers. Soc. Psychol. Rev. 8, 220–247 10.1207/s15327957pspr0803_1 15454347

[B28] Teachman B. A. Marker C. D. Smith-Janik S. B. (2008). Automatic associations and panic disorder: trajectories of change over the course of treatment. J. Consult. Clin. Psychol. 76, 988–1002 10.1037/a0013113 19045967 PMC2593734

[B29] Unsworth N. Heitz R. P. Schrock J. C. Engle R. W. (2005). An automated version of the operation span task. Behav. Res. Methods 37, 498–505 16405146 10.3758/bf03192720

[B30] Watson D. Friend R. (1969). Measurement of social-evaluative anxiety. J. Consult. Clin. Psychol. 33, 448–457 5810590 10.1037/h0027806

[B31] Weeks J. W. Heimberg R. G. Fresco D. M. Hart T. A. Turk C. L. Schneier F. R. (2005). Empirical validation and psychometric evaluation of the brief fear of negative evaluation scale in patients with social anxiety disorder. Psychol. Assess. 17, 179–190 10.1037/1040-3590.17.2.179 16029105

[B32] Westberg P. Lundh L. Jönsson P. (2007). Implicit associations and social anxiety. Cogn. Behav. Ther. 36, 43–51 10.1080/08037060601020401 17364651

[B33] Wiers R. W. Eberl C. Rinck M. Becker E. S. Lindenmeyer J. (2011). Retraining automatic action tendencies changes alcoholic patients' approach bias for alcohol and improves treatment outcome. Psychol. Sci. 22, 490–497 10.1177/0956797611400615 21389338

